# Re-sequencing and genetic variation identification of a rice line with ideal plant architecture

**DOI:** 10.1186/1939-8433-5-18

**Published:** 2012-07-24

**Authors:** Shuangcheng Li, Kailong Xie, Wenbo Li, Ting Zou, Yun Ren, Shiquan Wang, Qiming Deng, Aiping Zheng, Jun Zhu, Huainian Liu, Lingxia Wang, Peng Ai, Fengyan Gao, Bin Huang, Xuemei Cao, Ping Li

**Affiliations:** 1 State Key Laboratory of Hybrid Rice, Sichuan Agricultural University, Chengdu, 611130 China; 2 Rice Research Institute, Sichuan Agricultural University, 211 Huimin Road, 611130 Wenjiang, Sichuan China; 3 Key Laboratory of Crop Genetic Resources and Improvement, Ministry of Education, Sichuan Agricultural University, 625014 Ya’an, Sichuan China

**Keywords:** Rice, IPA, Re-sequencing, SNP, InDel, SV

## Abstract

**Background:**

The ideal plant architecture (IPA) includes several important characteristics such as low tiller numbers, few or no unproductive tillers, more grains per panicle, and thick and sturdy stems. We have developed an *indica* restorer line 7302R that displays the IPA phenotype in terms of tiller number, grain number, and stem strength. However, its mechanism had to be clarified.

**Findings:**

We performed re-sequencing and genome-wide variation analysis of 7302R using the Solexa sequencing technology. With the genomic sequence of the *indica* cultivar 9311 as reference, 307 627 SNPs, 57 372 InDels, and 3 096 SVs were identified in the 7302R genome. The 7302R-specific variations were investigated via the synteny analysis of all the SNPs of 7302R with those of the previous sequenced none-IPA-type lines IR24, MH63, and SH527. Moreover, we found 178 168 7302R-specific SNPs across the whole genome and 30 239 SNPs in the predicted mRNA regions, among which 8 517 were Non-syn CDS. In addition, 263 large-effect SNPs that were expected to affect the integrity of encoded proteins were identified from the 7302R-specific SNPs. SNPs of several important previously cloned rice genes were also identified by aligning the 7302R sequence with other sequence lines.

**Conclusions:**

Our results provided several candidates account for the IPA phenotype of 7302R. These results therefore lay the groundwork for long-term efforts to uncover important genes and alleles for rice plant architecture construction, also offer useful data resources for future genetic and genomic studies in rice.

**Electronic supplementary material:**

The online version of this article (doi:10.1186/1939-8433-5-18) contains supplementary material, which is available to authorized users.

## Findings

### Field performances of 7302R

**Figure 1:**
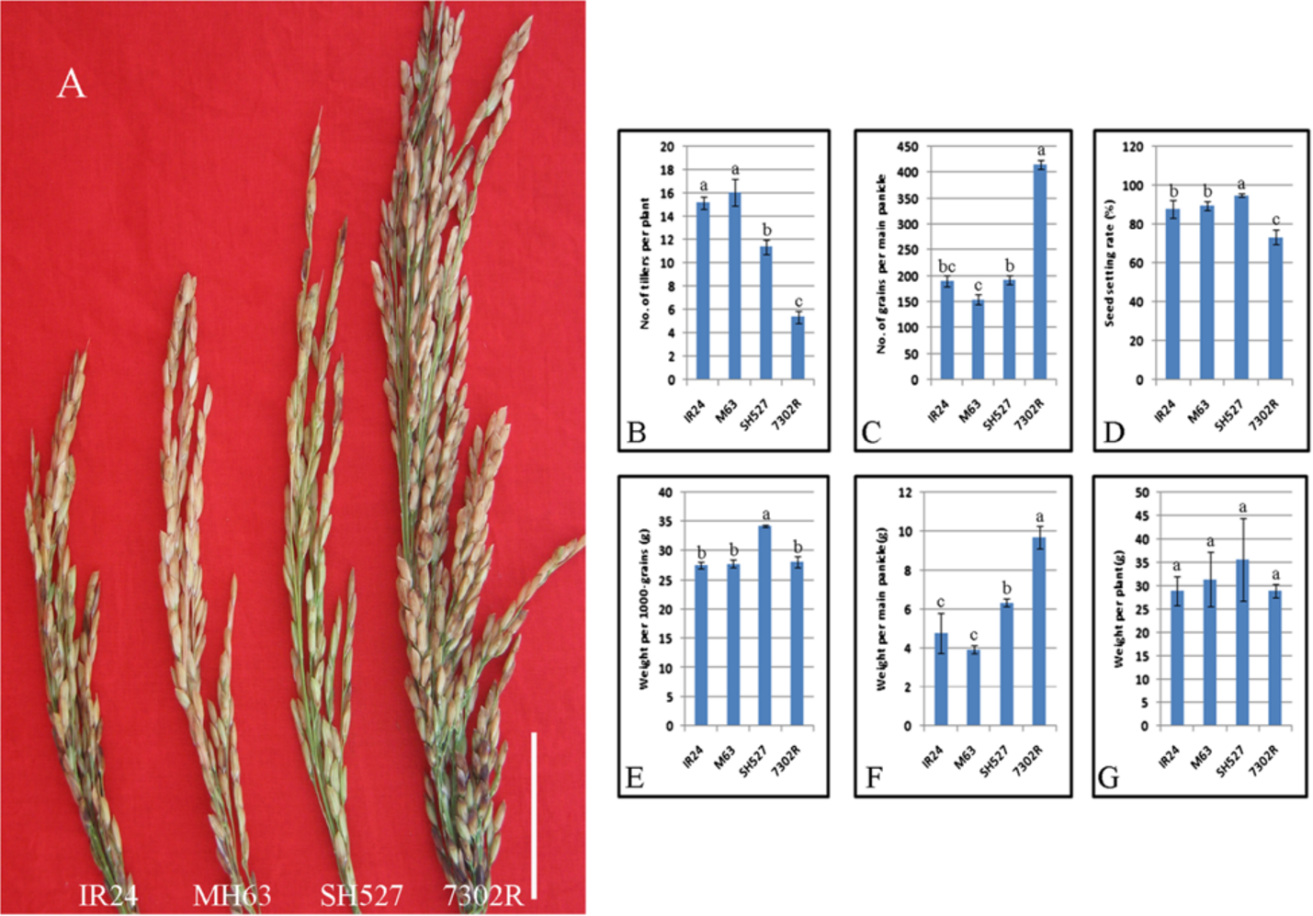
**Field performance of 7302R.** Comparison between the matured panicles of 7302R and of the other three core restorer lines (IR24, MH63, and SH527). The panicle of 7302R was apparently bigger than those of other lines (**A**). Comparison of the main yielding component traits, such as the number of tillers per plant (**B**), number of grains per main panicle (**C**), seed setting rate (**D**), weight per 1 000 grains (**E**), weight per main panicle (**F**), and weight per plant (**G**) between 7302R and the other lines (IR24, MH63, and SH527). Note that one-way ANOVA and LSD test using DPS Software were employed, and superscripts a, b, and c indicate the significant difference detected by the LSD test at *P* < 0.05.

We examined the field performances of 7302R (Figure 1) by comparing several of its yield-related traits with those of IR24, MH63, and SH527, which are considered as core representative restorer lines for hybrid rice. No obvious differences were found in the yield components of 1000 grain weight (Figure 1E) and weight per plant (Figure 1G) between 7302R and other lines. However, a significant decrease in the tiller number per plant (Figure 1B) and an increase in grain number per main panicle (Figure 1C) were observed. Moreover, the increase in grain number resulted in an apparent increase in the weight per main panicle (Figure 1F). Understandably, the seed setting rate of 7302R (Figure 1D) was decreased, which might have been a negative result of the huge increase in grain number. In addition, the stem of 7302R became stronger than those of the other rice lines. Upon comparison with other currently cultivated rice varieties, the newly developed *indica* restorer line 7302R was found to display the IPA phenotype, particularly in terms of tiller number, grain number, and stem strength.

### Genome sequencing and variation identification

The 7302R genotypes were determined with approximately a 10-fold coverage by genome sequencing using the Solexa sequencing technology. According to the protocol, a DNA library with an average insertion length of 484 bp was constructed and 6.35 G bases were generated. The alignment of reads was used to build consensus genome sequences for 7302R. Moreover, approximately 4.77 G high-quality raw databases were aligned with the reference sequence of cultivar 9311 using the SOAPaligner (Li et al. [2008]). An overall effective depth of 13× coverage was achieved (Table 1), and the resulting consensus sequence covered approximately 82.57% of the reference genome.

**Table 1:** **Summary of original sequencing data ( 9311 as the reference)**

Sample	Insert size	Bases (G)	Mapped bases (G)	Depth	Coverage (%)	Mismatch rate (%)
7302R	484	6.35	4.77	13.26	82.57	0.65

**Figure 2:**
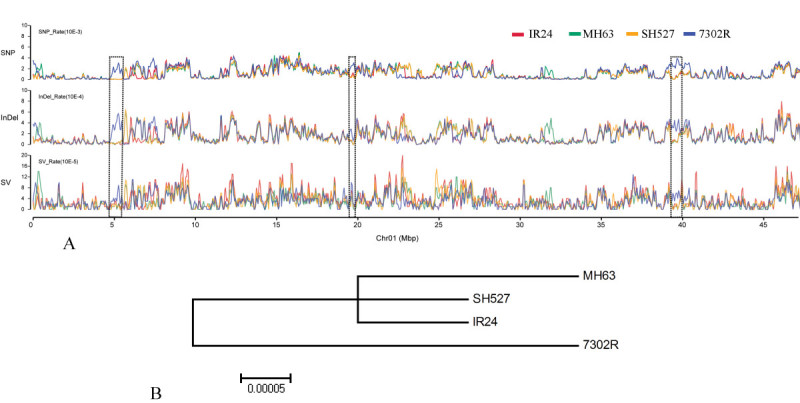
**Frequency distributions of variation and phylogenetic analysis.** (**A**) Frequency distribution comparisons of SNP, InDel, and SV of 7302R, IR24, MH63, and SH527 (chr. 1 for instance). The black dotted-line box indicates the SNP high region of 7302R covered by the InDel and SV high region. (**B**) Phylogenetic tree constructed by several authentic collections of SNPs, showing the relative relationship of the 7302R and other lines (IR24, MH63, and SH527).

Genome-wide variations were then examined via SOAPsnp11 and SOAPsv using a conservative quality filter pipeline (Li et al. [2009]), and 307 627 SNPs, 57 372 InDels, and 3 096 SVs were yielded from the 7302R genome (Table 2, Additional files 1,2 and 3). We have previously re-sequenced three important representative restorer lines, namely, IR24, MH63, and SH527, using the same technology (Li et al. [2012]). The overall genome diversity among these re-sequenced lines was much lower than that reported for a more diverse population (Huang et al. [2010]) because of the inherent relationship between these samples, suggesting a close relationship between the sequenced lines. The relative close relationship was also consistent with the previous result of restorer lines that have narrow genetic backgrounds (Duan et al. [2002]). A phylogenetic tree was constructed (Tamura et al. [2007]) using several authentic collections of SNPs for each sequenced line, and a relatively distant relationship was observed between 7302R and the other restorer lines (Figure 2B).

**Table 2:** **SNP, InDel and SV detected among the 7302R genome ( 9311 as the reference)**

Chr.	SNP	InDel	SV
Chr01	39,674	8,046	483
Chr02	36,901	7,199	231
Chr03	32,756	6,451	327
Chr04	27,159	4,810	282
Chr05	26,229	4,951	236
Chr06	24,092	4,424	225
Chr07	21,525	3,699	118
Chr08	27,935	5,200	421
Chr09	13,531	2,524	114
Chr10	22,382	3,826	253
Chr11	13,777	2,490	193
Chr12	21,666	3,752	213

The frequencies of SNPs, InDels, and SVs for 7302R were plotted at a 100 kb sliding window, with a step size of 50 kb along each chromosome, by comparing them with those of IR24, MH63, and SH527. The SNP/InDel/SV frequency was defined as the corresponding number of SNPs/InDels/SVs divided by the number of nucleotides within the 100 kb interval, excluding the uncovered nucleotides. Each sample was compared with the corresponding interval to identify regions that showed non-random variation frequencies (Figure 2A). Overall, 47/292, 91/124, and 39/571 SNP, InDel, and SV high/low regions were identified in the 7302R genome (Table 3). The most abundant chromosomes in the SNP high region were chr. 7, chr. 5, chr. 12, and chr. 2. Moreover, the most abundant chromosomes in the InDel high region were chr. 7, chr. 5, chr. 3, and chr. 2. Among these chromosomes, chr. 5 and chr. 2 were shown to be distributed with more SV high regions. These results show that these chromosomes are more abundant in genetic variations. In addition, several chromosomal loci in the SNP high region were also found covered by the InDel and SV high regions (Figure 2A), which might suggest that those regions were the most polymorphic.

**Table 3:** **High and low frequency regions of variation distribution in 7302R genome**

Chr.	SNP_high	SNP_low	InDel_high	InDel_low	SV_high	SV_low
Chr01	0	64	3	20	3	72
Chr02	6	13	19	9	8	49
Chr03	0	32	15	16	3	93
Chr04	3	32	2	5	3	71
Chr05	8	16	19	13	10	28
Chr06	5	32	6	11	4	65
Chr07	10	27	13	19	1	60
Chr08	1	14	2	6	2	23
Chr09	4	17	4	1	0	22
Chr10	1	12	4	2	2	17
Chr11	2	23	0	18	0	42
Chr12	7	10	4	4	3	29
Total	47	292	91	124	39	571

### Identification and characterization of 7302R-specific SNPs

**Figure 3:**
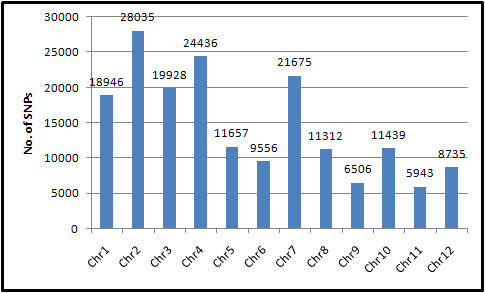
**7302R-specific SNP identification and its distribution on each chromosome.** 7302R-specific SNPs were identified for those loci whose nucleotides were similar to those in IR24, MH63, and SH527 but were different from that of 7302R. Numbers of the 7302R-specific SNPs on each chromosome were indicated.

As the differences between 7302R and those none-IPA type lines may reflect the genetic improvement of the IPA-type rice from the current none-IPA cultivars, an investigation of the 7302R-specific variations was therefore performed using a synteny analysis of all the SNPs of the 7302R compared with those of IR24, MH63, and SH527. We revealed a total of 178 168 7302R-specific SNPs across the whole genome, and the distribution in each chromosome is shown in Figure 3. The chr. 2, chr. 4, and chr. 7 were found to be the three most abundant chromosomes in the 7302R-specific SNPs.

The SNPs in the coding regions were analyzed to further understand the potential functional effects of the 7302R-specific SNPs. A total of 30 239 SNPs were located in the predicted mRNA regions, among which 4 946 were synonymous coding sequences (Syn CDS) and 8 517 were non-synonymous coding sequences (Non-syn CDS) (Table 4).

**Table 4:** **Annotations of gene located 7302R-specific SNPs**

Chr.	Syn_CDS	Non-syn_CDS	mRNA
Chr01	461	898	3,445
Chr02	733	1,169	4,710
Chr03	536	928	3,978
Chr04	562	1,032	3,131
Chr05	298	558	1,893
Chr06	385	579	2,247
Chr07	702	1,055	3,482
Chr08	265	409	1,637
Chr09	248	413	1,382
Chr10	278	585	1,632
Chr11	266	507	1,406
Chr12	212	384	1,296

**Figure 4:**
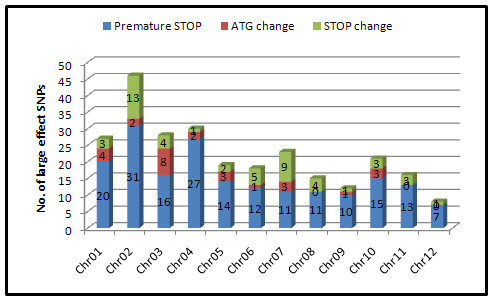
**Large-effect 7302R-specific SNP identification and its distribution on each chromosome.** A total of 263 large-effect SNPs were identified from the 7302R-specific SNPs. The numbers of its distribution and type on each chromosome were indicated.

Two hundred sixty-three large-effect SNPs that were expected to affect the integrity of the encoded proteins were further identified from the 7302R-specific SNPs (Figure 4). These included 187 premature terminations, 27ATG changes, and 49 stop changes. Be accordance with the distribution of 7302R-specific SNPs, Chr. 2, chr. 4, and chr. 7 were also the three most abundant large-effect SNP chromosomes.

**Figure 5:**
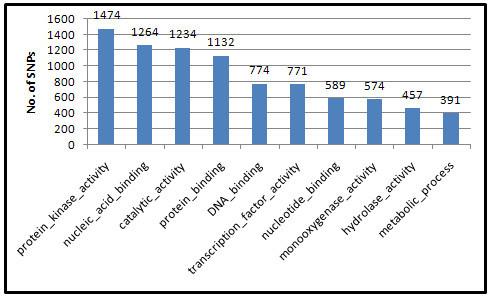
**Top 10 GOs of the 7302R-specific SNPs.** Each gene that has 7302R-specific SNP was functionally annotated with the GO annotation data, and the top 10 GOs were listed. The numbers of genes in each GO were indicated.

Gene Ontology analyses were further conducted for the genes in the 7302R-specific SNPs to explore the gene functions. Investigations showed that the top GOs were protein kinase activity, nucleic acid binding, catalytic activity, protein binding, and DNA binding (Figure 5). Our finding is partially consistent with our previous result of the gene function analysis of variations between restorer lines (Li et al. [2012]).

### Variation analysis on important rice genes

We investigated the natural variations among ~60 genes, which might explain the phenotypic differences of the sequenced sample. A large number of SNPs (Table 5) were detected both in the DNA sequence and in the coding regions of genes related to disease resistance, such as *Pib* (Wang et al. [1999]), *Xa1* (Yoshimura et al. [1998]), and *Xa21* (Song et al. [1995]). Other disease resistance genes, *such as Pi9* (Qu et al. [2006]), *Xa26 (* Sun et al. [2004]), *rTGA2.1* (Heather et al. [2005]), and *Pi-ta* (Bryan et al. [2000]), have at least one SNP in the predicted mRNA region. Our finding is also consistent with the previous result that genes mediating disease resistance in plants are particularly diverse due to pathogen pressure (Lai et al. [2010]). However, genes related to rice developmental processes, yield, and quality, such as *FLO4* (Kang et al. [2005])*, DEP1* (Huang et al. [2009])*, GS3* (Fan et al. [2006]; Mao et al. [2010])*, EUI1* (Zhu et al. [2006]), *Gn1a* (Ashikari et al. [2005]) and *qSW5* (Shomura et al. [2008]), had rare or no variations in the coding regions although they were found to have several SNPs in the DNA sequence. Especially, no variations were found in the *IPA1* (Jiao et al. [2010]) locus and might suggest the IPA phenotype of 7302R was not associated with the recently isolated rice architecture gene. Interestingly, a number of SNPs were found both in the DNA sequence (~15) and in the coding regions (~11) of *Rf1a* (Wang et al. [2006]), a possible allelic gene for *Rf4* (Ahmadikhah et al. Ahmadikhah and Karlov [2006]), which is the major restoring gene of the WA-CMS line. These variations may be due to the differences between the restoring abilities of 7302R and other sequenced restorer lines. This observation is consistent with and might well explain the restoring range and ability difference observed in the breeding practice between them. In the same way, the variations in the *SSIIIa* (Fujita et al. [2007]) DNA sequence (~19) and in the coding regions (~7) might have been responsible for the grain quality difference.

**Table 5:** **SNP Detection of cloned important rice genes**

Gene	DNA	mRNA
*ALK*	1	0
*Bph-14*	1	0
*DWARF10*	2	0
*DWARF 27*	2	0
*DEP1*	7	0
*EUI1*	4	0
*FLO4*	19	0
*GIF1*	1	0
*Gn1a*	1	0
*GS3*	6	0
*GW2*	2	0
*HTD2*	1	0
*OsGT1*	3	0
*Pi9*	1	1
*Pib*	25	14
*P-id2*	7	0
*Pi-ta*	8	2
*qSW5*	3	0
*Rf1a*	15	11
*rTGA2.1*	1	1
*sd1*	2	0
*OsSSIIIa*	19	7
*Xa1*	5	4
*Xa21*	11	7
*Xa26*	3	1
*Xa5*	9	0

In the present study, we report variations over the whole genome of a rice cultivar with an IPA phenotype. However, further analysis of more related lines is necessary to better understand the IPA mechanism although useful information have been proposed to account for the IPA phenotype. Several follow-up steps can also be taken to determine candidate genes that may contribute to this phenotype. The large-effect SNPs and known important rice gene-located SNPs should also be strictly selected and considered for functional verifications. Furthermore, we have developed several genetic populations with 7302R for the dissection and mapping of IPA components. The QTL mapping result and variation distributions are expected to make candidate fixing and further functional confirmation easy. The present study therefore lays the groundwork for long-term efforts to uncover genes and alleles important in rice plant architecture construction, also offers useful data resources for future genetic and genomic studies in rice.

## Electronic supplementary material

Additional file 1: **Data files are generally TXT. For Windows user, “Editplus” or “UltraEdit” is recommended as the browser program.** SNPs information of 7302R; Format description(left to right). 1. Chromosome name. 2. Position of locus. 3. Nucleotide at corresponding locus of reference sequence. 4. Genotype of sequencing sample. 5. Quality value. 6. nucleotide with the highest probability(first nucleotide). 7. Quality value of the nucleotide with the highest probability. 8. Number of supported reads that can only be aligned to this locus. 9. Number of all supported reads that can be aligned to this locus. 10. Nucleotide with higher probability. 11. Quality value of nucleotide with higher probability. 12. Number of supported reads that can only be aligned to this locus. 13. Number of all supported reads that can be aligned to this locus. 14. Total number of reads that can be aligned to this locus. 15. Order and quality value. 16. Estimated copy number for this locus. 17. Presence of this locus in the dbSNP database. 1 refers to presence and 0 refers to inexistence. 18. The distance between this locus and another closest SNP. (SNP 19 MB)

Additional file 2: **InDels information of 7302R; Format description(left to right).** 1. Chromosome. 2. Position. 3. Indel type and number. 4. Bases. 5. Strand (+, positive; -, negative; *, both positive and negative). 6. Homozygosis or heterozygosis. 7. Average quality. 8. The number of supported read pair. 9. The number of all crossed read pair. (INDEL 2 MB)

Additional file 3: **SVs information of 7302R; Format description(left to right).** 1. Chromosome name. 2. Type of structure variation. 3. Minimal value of start position in cluster. 4. Maximal value of end position in cluster. 5. Estimated start position of this structure variation. 6. Estimated end position of this structure variation. 7. Length of SV. 8. Breakpoint of SV (only for insertion). 9. Unusual matching mode (F refers to align with forward sequence, R refers to align with reverse sequence). 10. Number of paired-end read which support this structure variation. (SV 233 KB)

Below are the links to the authors’ original submitted files for images.

Authors’ original file for figure 1

Authors’ original file for figure 2

Authors’ original file for figure 3

Authors’ original file for figure 4

Authors’ original file for figure 5

Authors’ original file for figure 6

## Abbreviations

IPA: Ideal plant architecture
SNP: Single-nucleotide polymorphisms
InDel: Insertion/deletion polymorphisms
SV: Structural variations
Non-syn CDS: Non-synonymous coding sequences.
