# Supplementary figures and images for: Re-sequencing and genetic variation identification of a rice line with ideal plant architecture
Source: Rice (N Y). 2012 Jul 24;5:18. doi: 10.1186/1939-8433-5-18 (PMC5520836; doi:10.1186/1939-8433-5-18)

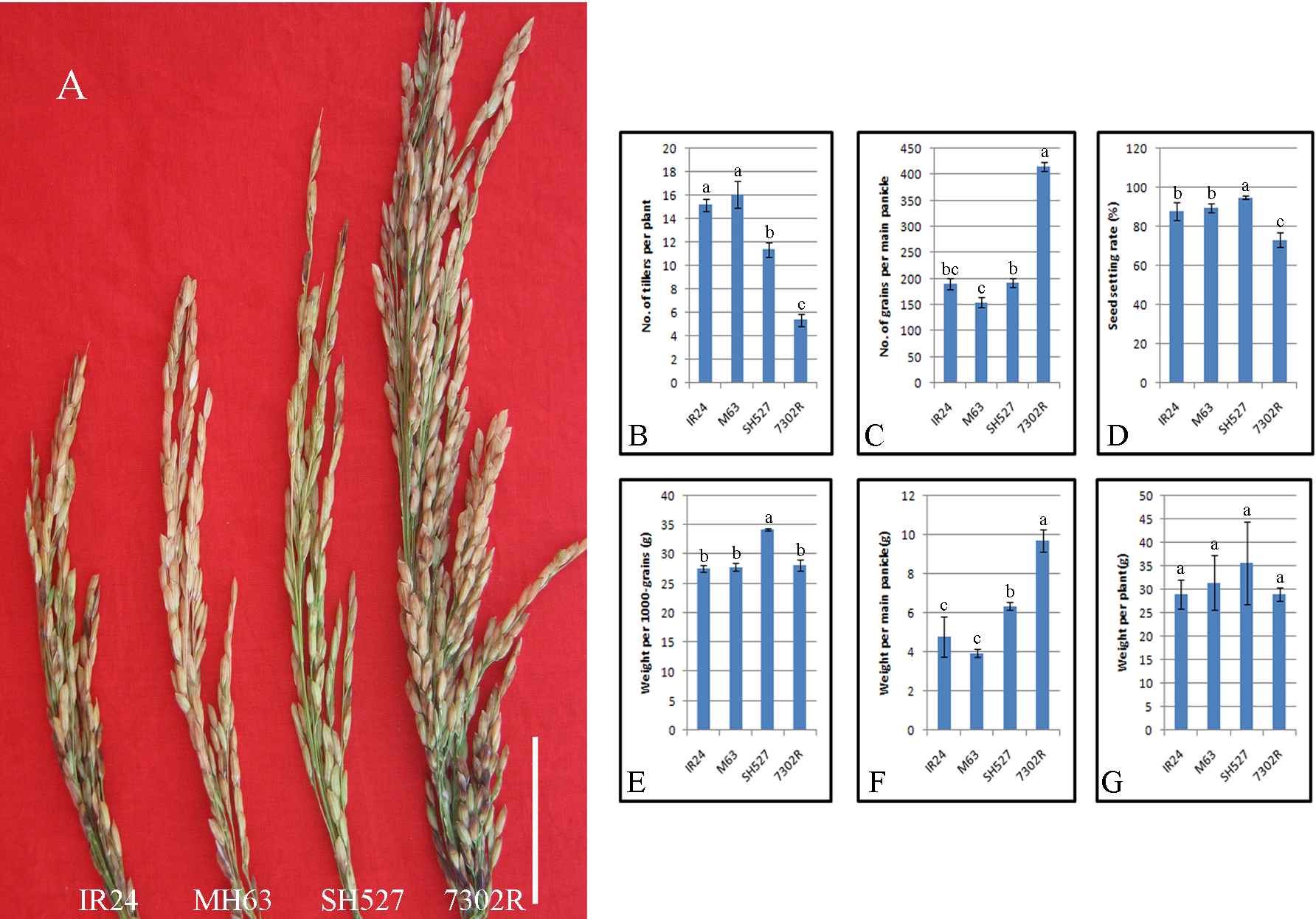

Supplement: Supplementary file 4 — Authors’ original file for figure 1 [file 12284_2012_13_MOESM4_ESM.tiff]

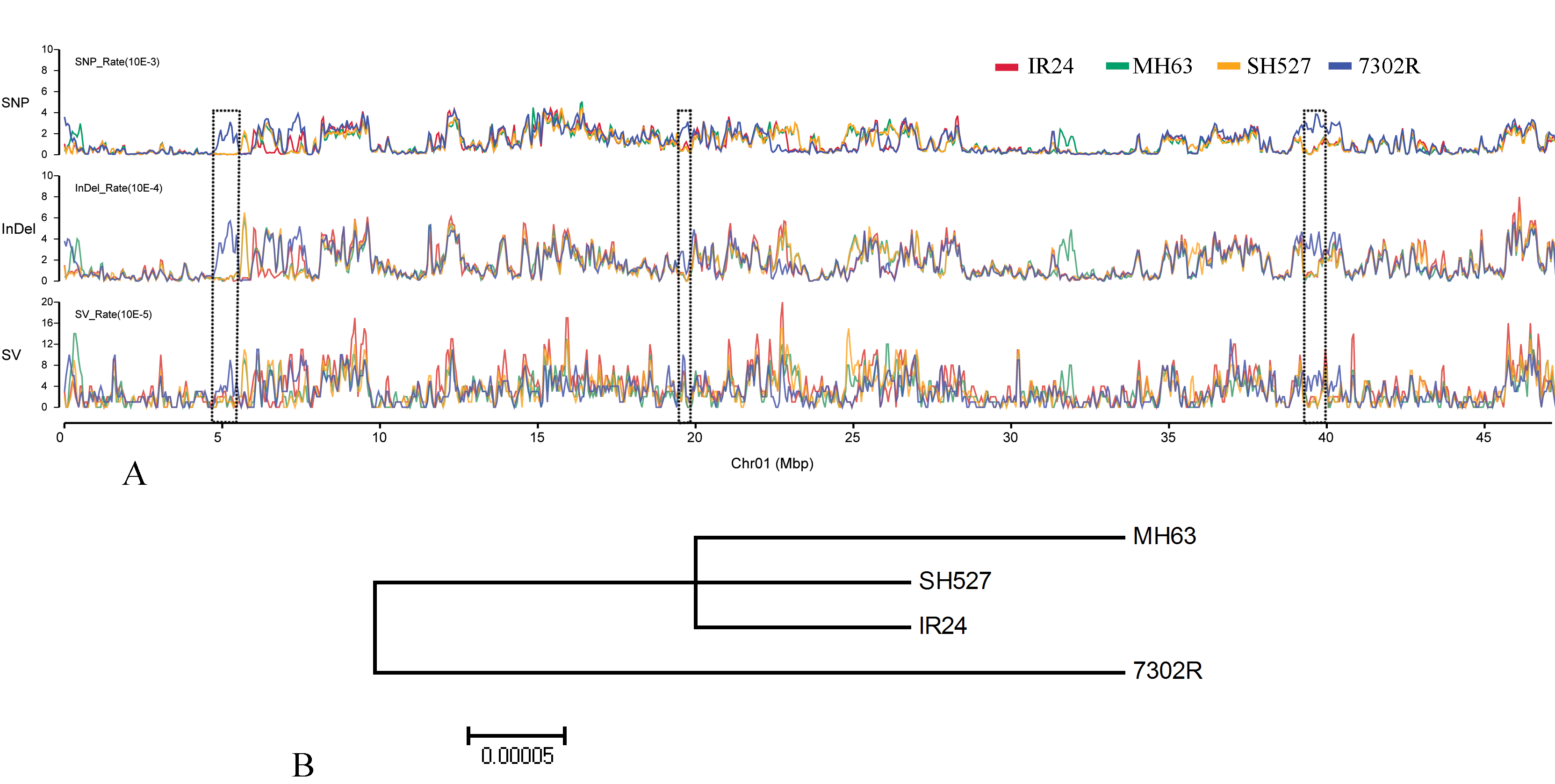

Supplement: Supplementary file 5 — Authors’ original file for figure 2 [file 12284_2012_13_MOESM5_ESM.tiff]

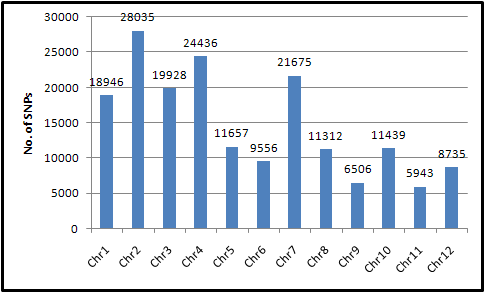

Supplement: Supplementary file 6 — Authors’ original file for figure 3 [file 12284_2012_13_MOESM6_ESM.tiff]

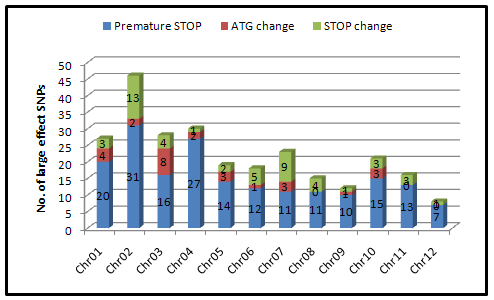

Supplement: Supplementary file 7 — Authors’ original file for figure 4 [file 12284_2012_13_MOESM7_ESM.tiff]

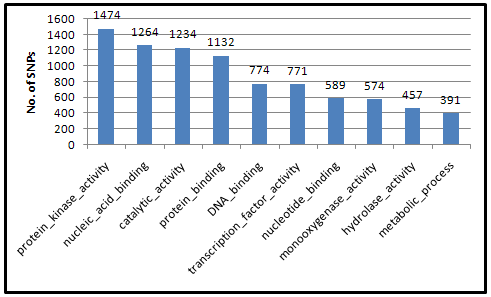

Supplement: Supplementary file 8 — Authors’ original file for figure 5 [file 12284_2012_13_MOESM8_ESM.tiff]
